# Quantitative structure–activity relationship models for genotoxicity prediction based on combination evaluation strategies for toxicological alternative experiments

**DOI:** 10.1038/s41598-021-87035-y

**Published:** 2021-04-13

**Authors:** Xiaotong Yang, Zhengbao Zhang, Qing Li, Yongming Cai

**Affiliations:** 1 School of Public Health, Guangdong Pharmaceutical University, Guangzhou, China; 2 Guangdong Province Center for Disease Control and Prevention, Guangzhou, China; 3 College of Medical Information Engineering, Guangdong Pharmaceutical University, Guangzhou, China; 4 Guangdong Provincial TCM Precision Medicine Big Data Engineering Technology Research Center, Guangzhou, China

**Keywords:** Computational biology and bioinformatics, Genetics, Chemistry, Mathematics and computing

## Abstract

Mutagenicity exerts adverse effects on humans. Conventional methods cannot simultaneously predict the toxicity of a large number of compounds. Most mutagenicity prediction models are based on a single experimental type and lack other experimental combination data as support, resulting in limited application scope and predictive ability. In this study, we partitioned data from GENE-TOX, CPDB, and Chemical Carcinogenesis Research Information System according to the weight-of-evidence method for modelling. In our data set, in vivo and in vitro experiments in groups as well as prokaryotic and eukaryotic cell experiments were included in accordance with the ICH guideline. We compared the two experimental combinations mentioned in the weight-of-evidence method and reintegrated the experimental data into three groups. Nine sub-models and three fusion models were established using random forest (RF), support vector machine (SVM), and back propagation (BP) neural network algorithms. When fusing base models under the same algorithm according to the ensemble rules, all models showed excellent predictive performance. The RF, SVM, and BP fusion models reached a prediction accuracy rate of 83.4%, 80.5%, 79.0% respectively. The area under the curve (AUC) reached 0.853, 0.897, 0.865 respectively. Therefore, the established fusion QSAR models can serve as an early warning system for mutagenicity of compounds.

## Introduction

In genetics, a mutagen is a physical or chemical agent that changes the genetic material, usually DNA, of an organism and thus increases the frequency of mutations above the natural background level. Mutagens are genotoxic and can affect or dysregulate the molecular central dogma process, namely replication, transcription, and translation. Some mutagens can dysregulate cell proliferation and cell death, thus causing cancer. Therefore, the detection of compound mutagenicity of great significance.

Various experimental methods have been developed to detect mutagenic toxicity based on the different action mechanisms of mutagenicity and the key points of mutagenic detection. Conventional mutagenicity tests include sex-linked recessive lethal test, dominant lethal test, and mammalian bone marrow cytogenetics test. These tests can also be followed by Ames test and comet assay for mutagenicity detection. However, because the determination of mutagenicity of compounds is complex, no single experimental method can detect all mutagenic mechanisms. Generally, the results of in vivo experiments, in vitro experiments, prokaryotic cell experiments, and eukaryotic cell experiments must be included. There are so many types of detection experiments that it is unrealistic to predict each type of experiment separately. Therefore, these types of experiments need to be combined according to reasonable rules.

The International Council for Harmonisation of Technical Requirements for Pharmaceuticals for Human Use (ICH) recommends two combinations of compound mutagenicity test^1^: *Option 1: i. Bacterial reverse mutation test (Ames); ii. Mammalian erythrocyte micronucleus test or mammalian bone marrow cell chromosome aberration test; iii. *in vitro* mammalian cell chromosomal aberration test or *in vitro* mammalian cell TK gene mutation test. Option 2: i. Bacterial reverse mutation test (Ames); ii. mammalian erythrocyte micronucleus test or mammalian bone marrow cell chromosome aberration test; iii. mammalian spermatocyte chromosome aberration test or rodent dominant lethal test.*

Only few studies have adopted the combined method recommended by the ICH to study the mutagenicity of compounds. For example, Kasamoto et al. validated an in vivo comet-micronucleus combination assay in rats^2^. In addition, in their mutagenicity study of tricyclazole, Corvaro et al. conducted both in vivo and in vitro experiments and obtained a strong evidence that: the detection effectiveness of a single experiment is one-sided, and it is not enough to determine whether the compound is toxic. Only by comprehensively considering multiple assays in combination can a correct conclusion be reached^3^.

However, in general, the number of studies employing experiment combinations were relatively small, and all these studies tested only one compound. Owing to the shortcomings of conventional experiments, including long cycle, high cost, and ethical problems of experimental animals, it is difficult to meet the need for massive testing of compounds. Consequently, computational methods for predicting the mutagenicity of compounds have gained attention to overcome these shortcomings^4, 5^. Among the experimental types used for prediction via computational models, Ames mutagenicity test has shown more and more successful predictions. For example, the QSAR model developed by Honma et al., which is based on the largest Ames dataset, can reach a prediction accuracy of more 80%^6^. Moreover, some Ames prediction models can also obtain relatively good prediction results^7–9^. In addition, other studies focused on partial genetic toxicity endpoint predictions for a single class of compounds^10–12^.

However, prediction based on single experimental endpoint is flawed. For example, some mutagenic compounds can show negative Ames test result; thus, the model will make wrong predictions. Therefore, to overcome the deficiency of the existing prediction models and to achieve both the evaluation ability of combined experiments and the efficiency of computational methods, there is an urgent need to establish a database integrating multi-type mutation experimental data, and then establish a fusion model for prediction based on this database.

To this end, in this study, we collected mutagenic experimental results of the same compound from three databases, which covered all experimental types in the experimental combination mentioned in the ICH guidelines. Different mutagenic experimental combinations of the same compound were used to establish sub-models, and the predicted output values of three sub-models under the same algorithm were used as the input values of the fusion model in this study. The criterion was “all-negative is judged as negative, otherwise positive”. Under this fusion strategy, the ensemble model showed excellent and robust prediction performance. It is thus expected to be a sentinel model for accurately predicting the mutagenicity of compounds.

## Results

### Combination of mutagenic experimental data and analysis of data sets

After grouping the results of multiple mutagenic experiments of the same compound according to the weight-of-evidence principle, we obtained three groups of independent mutagenic experimental results: Y1, Y2, and Y3, which are shown in Table 1.

**Table 1:** Experiments in two combinations according to weight-of-evidence method.

	Y1	Y2	Y3
Combination 1	Bacterial reverse mutation test (Ames)	Mammalian erythocytel micronucleus test/mammalian bone marrow cell chromosome aberration test	In vitro mammalian cell chromosomal aberration test/in vitro mammalian cell TK gene mutation test
Combination 2	Bacterial reverse mutation test (Ames)	Mammalian erythocyte micronucleus test/mammalian bone marrow cell chromosome aberration test	Mammalian spermatocyte chromosome aberration test/rodent dominant lethal test

A total of 665 compounds were divided into training and test sets at a ratio of 4:1, containing 532 and 133 compounds, respectively. The experimental data distribution of Y1, Y2, and Y3 is shown in Table 2.

**Table 2:** The experimental data distribution of Y1, Y2, and Y3.

	Y1		Y2		Y3	
	+	−	+	−	+	−
Training set	230	302	296	236	282	250
Test set	53	80	76	57	74	59
Total	283	382	372	293	356	309

In the training set, the positive rate of Y1 (Ames experiment), Y2, and Y3 was 43.23%, 55.64%, and 53.01%, respectively. In the test set, the positive rate of Y1, Y2, and Y3 was 39.85%, 57.14%, and 55.64%, respectively.

### Selection of molecular descriptors

**Figure 1:**
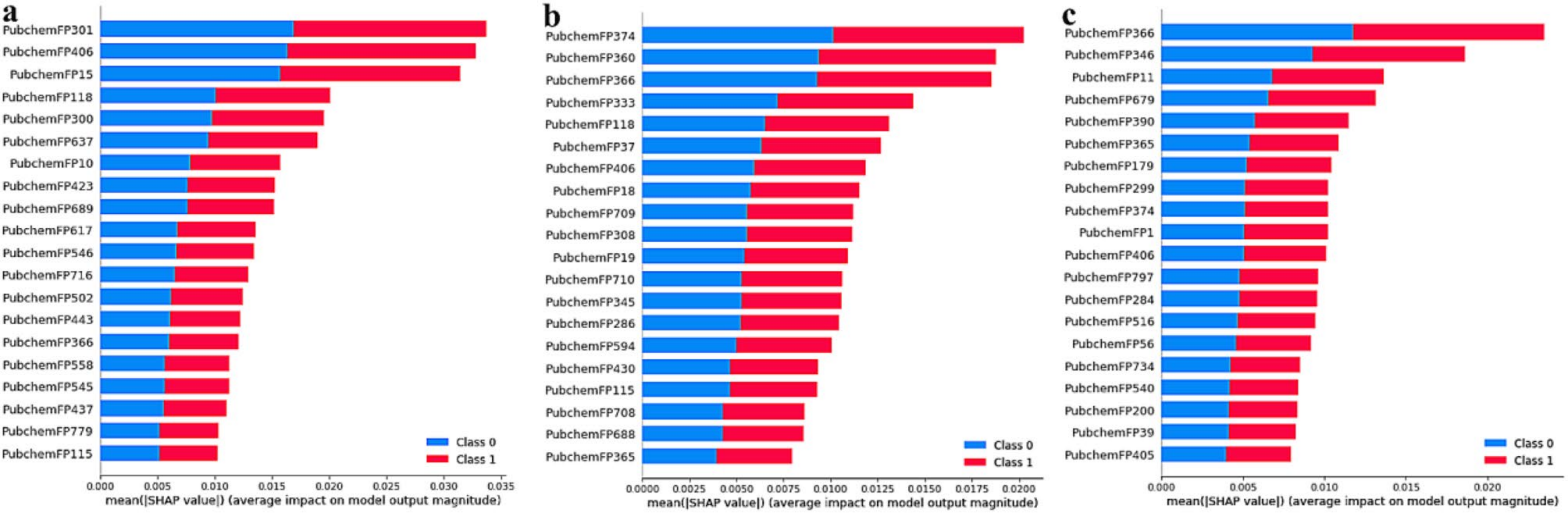
Top 20 Pubchem fingerprints’ SHAP value of each three sets of experiments. (**a**. shows the first 20 SHAP value of the Pubchem fingerprints corresponding to the experimental outcome of the group Y1; **b**. shows the first 20 SHAP value of the Pubchem fingerprints corresponding to the experimental outcome of the group Y2; **c**. shows the first 20 SHAP value of the Pubchem fingerprints corresponding to the experimental outcome of the group Y3).

881 Pubchem sub-structure fingerprints^13^ were calculated to characterize the structure of the compound and used as features for the next step of screening. Three sets of SHAP SHapley Additive exPlanations (SHAP) values^14^ of each feature were calculated and sorted from largest to smallest, and the intersection of the three sets was included as descriptors in the final model. Figure 1 show SHAP values with three sets of experiments as dependent variables; the top 20 molecular descriptors were displayed from large to small.

After full testing and demonstration, the best model performance was obtained when the intersection was taken within the first quintile of the three sets of SHAP values. The final 89 key molecular fingerprints were displayed in the supplementary materials and arranged in descending order according to their SHAP values.

### Modelling and determination of compound mutagenicity

The experimental outcome data of all compounds in this study were positive or non-positive dichotomous. The positive outcome was expressed by value 1, whereas the non-positive outcome was expressed by value 0. The specific modelling strategy and flow chart of this study are shown in “Methods” section.

First, random forest (RF), support vector machine (SVM), and back propagation (BP) neural network algorithms were used to predict three sets of mutagenic attributes of compounds, respectively.

Next, according to the weight-of-evidence principle, the sub-models of each algorithm were fused to obtain the final result. The rule of mutagenicity judgement in this study was that when all the experimental groups were negative, the outcome was negative, otherwise it was positive. Therefore, to achieve effective model prediction and control overfitting, the predicted output values of three sub-models under the same algorithm were used as the input values of the fusion model. Under this rule of fusion, each ensemble model achieved the expected excellent effect after merging the sub-model.

Through fivefold cross-validation, the fusion model showed that the accuracy of the three algorithms (RF, SVM, BP neural network algorithm) for mutagenicity prediction reached 83.4%, 80.5%, and 79.0%, respectively, AUC value reached 0.853, 0.897, 0.865respectively. Table 3. shows the predictive accuracy comparison of different models in the training and test sets.

**Table 3:** Comparison of prediction accuracy calculated through fivefold cross-validation in different model’s training sets and test sets.

Algorithm	Sub-model						Ensemble model	
	Y1		Y2		Y3		All groups	
	Training set	Test set	Training set	Test set	Training set	Test set	Training set	Test set
RF	0.858	0.685	0.768	0.635	0.776	0.623	0.825	0.805
SVM	0.725	0.595	0.797	0.587	0.805	0.540	0.784	0.790
BP nueral network	0.878	0.690	0.834	0.580	0.891	0.610	0.872	0.795

### Model validation

Model validation includes internal and external validation. The internal verification mainly tests the fitness and robustness of the model, and the external test mainly tests the predictive ability of the model. The main indicators in this study include accuracy, precision, recall, F1-Measure, and AUC.

Within the range of the training set, the fivefold cross-validation method was chosen to test the robustness of the model. After testing, the prediction accuracy rates of the nine models of the three algorithms were 85.8%,76.8%, and 77.6%; 72.5%, 79.7%, and 80.5%; 87.8%, 83.4%, and 89.1%. Taken together, all three algorithms showed good fitness and robustness.

**Figure 2:**
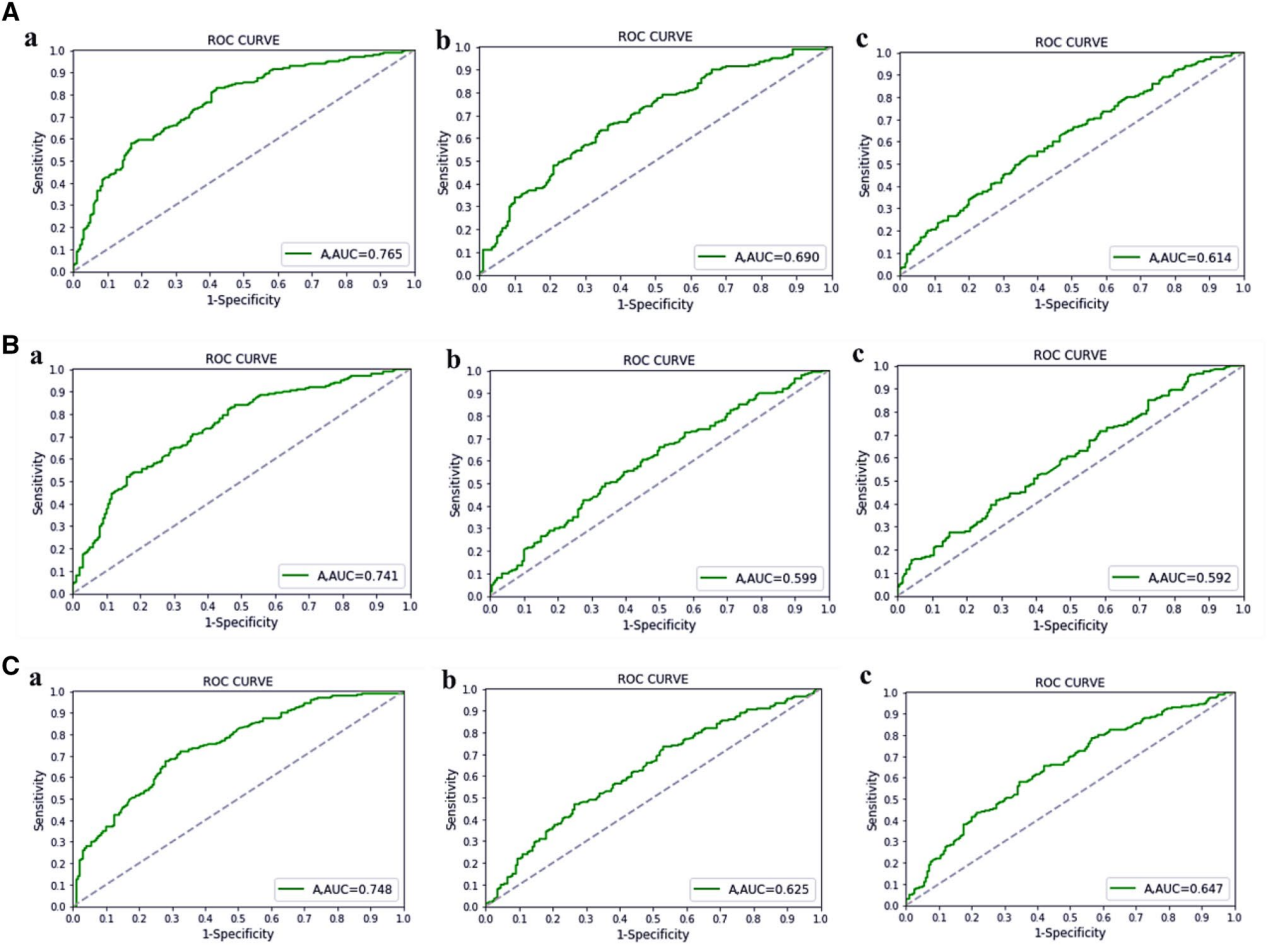
(**A**) ROC curves of the three sub-models of the RF algorithm (Figures a, b, and c respectively correspond to the ROC curves of the sub-models of groups Y1, Y2, and Y3). (**B**) ROC curves of the three sub-models of the SVM algorithm (Figures a, b, and c respectively correspond to the ROC curves of the sub-models of groups Y1, Y2, and Y3). (**C**) ROC curves of the three sub-models of the BP neural network algorithm (Figures a, b, and c respectively correspond to the ROC curves of the sub-models of groups Y1, Y2, and Y3).

External verification of the model was carried out within the test set. The overall prediction accuracy was 68.5%, 63.5%, and 62.3% for the RF algorithm; 59.5%, 58.7%, and 54.0% the SVM algorithm; and 69.0%, 58.0%, and 61.0% for the BP neural network algorithm. The corresponding AUC values were 0.765, 0.690, and 0.614; 0.741, 0.599, and 0.592; and 0.748, 0.625, and 0.647, respectively. The other main index values are shown in Table 4. The AUC of the test set is shown in Fig. 2.

**Table 4:** Model verification indicators for nine sub-models. (The positive and negative signs indicate the status of each indicator when the result is positive or non-positive).

Algorithm	Group	Recall		Precision		F1		Accuracy	AUC
		+	−	+	−	+	−		
RF	Y1	0.481	0.824	0.650	0.700	0.553	0.756	0.685	0.765
	Y2	0.796	0.425	0.643	0.617	0.711	0.503	0.635	0.690
	Y3	0.601	0.524	0.613	0.611	0.690	0.556	0.623	0.614
SVM	Y1	0.233	0.972	0.741	0.643	0.354	0.746	0.595	0.741
	Y2	0.782	0.519	0.613	0.539	0.761	0.410	0.587	0.599
	Y3	0.669	0.469	0.613	0.526	0.701	0.519	0.540	0.592
BP nueral network	Y1	0.605	0.748	0.620	0.736	0.613	0.742	0.690	0.748
	Y2	0.487	0.701	0.679	0.513	0.567	0.592	0.580	0.625
	Y3	0.556	0.674	0.667	0.563	0.606	0.614	0.610	0.647

The nine models established by the three algorithms in this study all showed good internal verification effects, and their fitness and robustness indicators were all above 72%. In the overall prediction effect of the model, the performance of the three algorithms was different.

**Figure 3:**
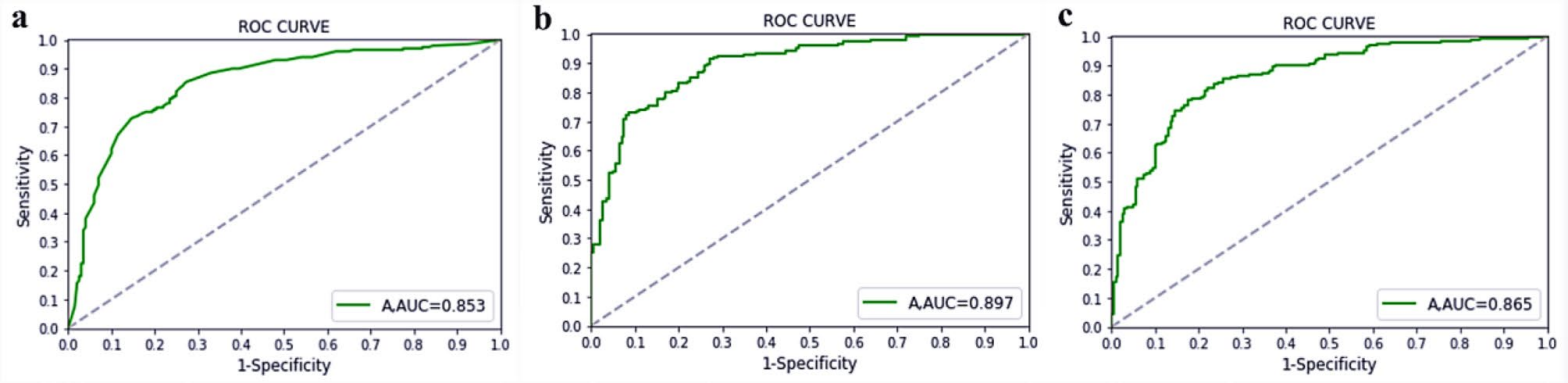
The ROC curve of the fusion model under the three algorithms. (The following three figures in sequence are the ROC curve of RF; the ROC curve of SVM and the ROC curve of BP neural network).

Through fivefold cross-validation, the fusion model showed that the accuracy of the three algorithms for mutagenicity prediction reached 82.5%, 74.8%, and 87.2%, respectively. The performance indicators of the fusion model under the three algorithms are shown in Table 5. This result showed that compared with a single prediction model, the fused model can predict compound mutagenicity in a more effective and stable manner. The prediction performance of the fusion model achieved good results in both the training and test sets, and the fusion of the sub-models alleviates the over-fitting phenomenon that occurs on a single sub-model. The receiver operating characteristic (ROC) curve of the fusion model is shown in Fig. 3.

**Table 5:** Indicators of fusion models through fivefold cross validation.

Algorithm	Recall		Precision		F1		Accuracy	AUC
	+	−	+	−	+	−		
RF	0.930	0.190	0.812	0.421	0.876	0.262	0.834	0.853
SVM	1	0.071	0.802	1	0.133	0.890	0.805	0.897
BP	0.914	0.732	0.790	0.115	0.883	0.103	0.790	0.865

## Discussion

For QSAR models, data quality and selection of molecular descriptors are highly important, particularly when building a classification prediction model. In this study, we compiled data on compound mutagenicity from three authoritative public databases, and after careful comparison and statistics according to the standards, the structure information of the corresponding compounds was supplemented to form a complete data set of compound structures and mutagenicity experiments. Based on this compound structure and mutagenicity test data set, a high-efficiency QSAR prediction model was established.

However, in comparison, there was a certain gap in the model’s prediction performance between the training and test sets. We noticed that regardless of the algorithm, for group Y1, which was the predictive index of the Ames experimental group, almost all sub-modes showed a phenomenon in which the predictive index value of positive results was lower than that of negative results. For group Y2, whose experimental results included mammalian red blood cell micronucleus test or mammalian bone marrow cell chromosomal aberration test, the predictive effect on positive results was slightly better than negative that on results. In fact, owing to methodological weaknesses, certain chemical substances with positive or negative experimental results may be incorrectly classified in advance, which led to false positives or false negatives in the QSAR model prediction. These erroneous data hindered prediction, reduced the prediction effectiveness of the model, and became a noise source in the QSAR model^6^. On the contrary, Y3 covered various in vitro experiments, which may be a reason for the decrease in the performance of the prediction model. At the same time, owing to limited access to the standards, some compound information was inevitably lost in this study.

As mentioned in “Methods” section, the prediction values of the output of the sub-model were used as the input values of the fusion model. The result showed that under this fusing strategy, the overfitting problem in the sub-model was resolved, and the generalisation ability and external prediction efficiency of the model were greatly improved through the fivefold cross-validation. This finding is of great significance for establishing a more comprehensive method to determine the mutagenicity of compounds. However, the lack of high-quality authoritative data is still a challenge in predicting compound toxicity.

In summary, in this study, we successfully established a QSAR model of compound mutagenicity, and the fusion model showed improved prediction performance compared with the single prediction model. The QSAR model established in this study, especially the fusion models, performed well in predicting positive results and can identify potential health hazards. These models can play a sentinel role in compound mutagenicity detection and become an early warning system. In the future, with the continuous accumulation of new compound information and related mutagenicity experimental data, the comprehensive database will continue to be enriched, and the predictive effectiveness of the QSAR model will also be improved.

## Methods

Computer technology was used for QSAR modelling to predict the mutagenicity of compounds. After the model was validated, the model was fused. When one or more sub-models in the combination were positive, the compound was considered mutagenic, and if all the sub-models in the combination were negative, the compound was judged to be non-mutagenic.

### Acquisition and integration of experimental data sets on mutagenicity

There are various tests for determining the mutagenicity of compounds, each with different detection endpoints and effects. Generally, the results of in vivo experiments, in vitro experiments, prokaryotic cell experiments, and eukaryotic cell experiments must be included when judging mutagenicity. Therefore, the International Council for Harmonisation criteria^1^ has developed a guideline for a combination of experiments that meets this principle for researchers' reference.

The content of the combination according to the ICH criteria are: Combination 1: Bacterial reverse mutation test (Ames), mammalian erythrocyte micronucleus test or mammalian bone marrow cell chromosome aberration test, in vitro mammalian cell chromosomal aberration test or in vitro mammalian cell TK gene mutation test. Combination 2: Bacterial reverse mutation test (Ames), mammalian erythrocyte micronucleus test or mammalian bone marrow cell chromosome aberration test, mammalian spermatocyte chromosome aberration test, or rodent dominant lethal test.

In this study experimental data on mutagenicity were obtained from three databases: Genetic Toxicology Data Bank (GENE-TOX), Carcinogenic Potency Database (CPDB), and Chemical Carcinogenesis Research Information System (CCRIS). They are all archived sub-databases of ToxNet and are no longer updated. The CPDB and GENE-TOX databases contain 1574 and 3214 compounds, respectively. The final data set comprised compound data from the two databases and data of experimental outcomes from the CCRIS database.

**Figure 4:**
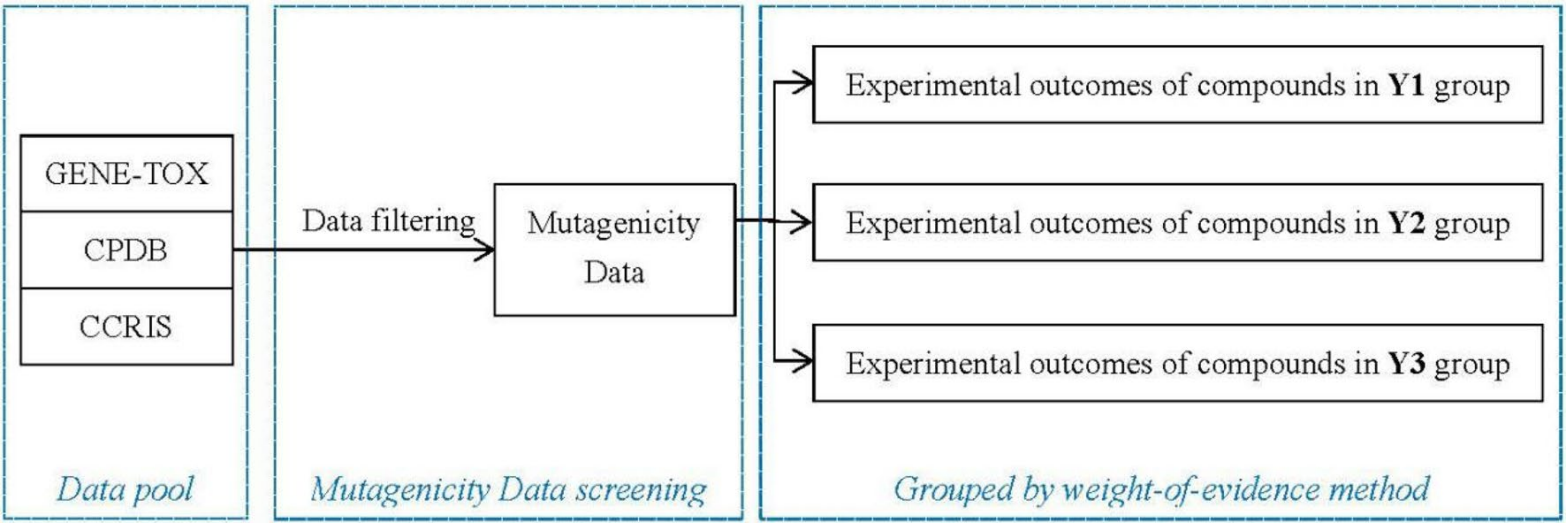
The flowchart of data collection and grouping processes.

The data collection and grouping processes for this study are shown in Fig. 4.

When there were missing data in the combination, to make optimal use of the available experimental data, other alternative experimental data, including in vitro mammalian cell HGPRT gene mutation experiment, in vitro mammalian cell DNA damage repair (unscheduled DNA synthesis) experiment, and *Drosophila*-associated recessive lethal experiment, were used as supplements according to the weight-of-evidence principle.

### Pre-processing of mutagenicity experiment results

For pre-processing, the experimental results were sorted into negative and positive dichotomous data, and only compounds with inconclusive experimental data were eliminated. We can find in Table 1 that the Y1 group represents the Ames test; the Y2 group represents the Mammalian erythocytel micronucleus test experiment or the mammalian bone marrow cell chromosome aberration test (they can be substituted for each other when the data is insufficient); the Y3 group covers a variety of experiments such as In vitro mammalian cell chromosomal aberration test, etc. (they also can be substituted for each other when the data is insufficient). In order to keep the data sets used for training the model tidy, we unified the results by voting when the Y2 and Y3 groups contain multiple experimental outcomes at the same time. When the number of results of two opposite experiments was equal, the compound was judged to be positive for the purpose of improving the sensitivity of the model.

For grouping of experimental data, the results of the two combinations were divided into three parts according to the type of experiment and the detection endpoint, and as a result, some experiments showed substitutability. Therefore, the results of the experimental combination were grouped as Y1, Y2, and Y3. Table 1 shows that Y1 and Y2 experimental types corresponded to combinations 1 and 2, whereas Y3 can be supplemented within the scope of combination 1 and combination 2 if there were insufficient data. Finally, 665 compounds without missing data in the three groups were obtained.

### Acquisition and calculation of structure descriptors

From the latest information in the three databases, information on compound name, CAS number, smiles code, and InChl code was compared to ensure that the collected data had no duplicate or error values. Finally, a compound structure information database was formed.

Molecular descriptors were used to characterise the molecular structure of the compounds. The chemical information was transformed into a digital form by logical or mathematical methods. Pubchem fingerprint covers a wide range of different substructures and features with 881 structural keys^13^. Models based on such descriptors can discover the relationship between compound structure and its mutagenicity more effectively.

PaDEL-Descriptor is a Java-based descriptor computing software that computes 1875 molecular descriptors (1444 1D/2D, 431 3D) and 12 molecular fingerprints^15^. The smiles codes of these compounds were imported into the PaDEL-Descriptor (version 2.21) software^16^ in batches, and the index values of 881 Pubchem fingerprints were calculated.

### Screening of molecular descriptors

Because there are many molecular descriptors that can be calculated by software, the selection of molecular descriptors needs to follow the minimum number principle, which can represent sufficient information. In general, the number of descriptors selected should not exceed one-fifth of the sample size^17^. In fact, even when the number of individuals in the sample increases further, the number of variables should not be increased.

SHapley Additive exPlanations (SHAP) values interpret the impact of having a certain value for a given feature in comparison with the prediction we would make if that feature took some baseline value. The advantage of a SHAP value is that it can reflect the influence of each characteristic on the result and show a positive or negative influence^14^.

Because of the particularity of the data in this study, experimental results were collected according to the experimental combination. Therefore, it was necessary to calculate the SHAP values of the descriptors that affect Y1, Y2, and Y3 separately.

After obtaining the three sets of SHAP values of the 881 fingerprints, they were sorted from largest to smallest, and the intersection of the three sets in a certain range from largest to smallest was the descriptor that was finally incorporated into the model. The final selected molecule descriptors are provided in “Results” section.

### QSAR model

This study mainly uses the RF, SVM, and BP neural network algorithm to establish a QSAR classification model. In the modelling process, the grid search algorithm was used to automatically find the optimal parameter combination of the model. When fusing sub-models, the predicted output values of sub-models under the same algorithm were used as the input values of the fusion model.

#### Selection of training and test set

Considering that the amount of compound data collected in this study was sufficient and the distribution was relatively balanced, the random selection method was selected to divide the dataset into training and test sets at a ratio of 7:3.

#### RF classification model

The RF algorithm is an ensemble learning method of multiple decision tree units^18^. In this study, the RF algorithm was used to model and predict the three groups of experimental data: Y1, Y2, and Y3. All steps were implemented in Python (version 3.7) using the scikit-learn (Vision 0.19.1) machine learning package^19^.

The model was evaluated and iterated by the Gini coefficient, and the prediction result was then generated according to the weight-of-evidence principle, “one positive result leads to a final positive result; only when all results are non-positive can the final judgement be non-positive”. Thus, the three prediction results were integrated to form the final judgement of compound mutagenicity.

#### SVM classification model

To build a better model, the corresponding parameter combination was adjusted, the appropriate kernel function was selected, and the parameters of the kernel function, penalty coefficient C, and size of ε in the ε-insensitive loss function were determined. Owing to the large correlation between these parameters, this study used the grid search (GridSearchCV) method to arrange and combine the possible values of each parameter. After the fitting function had tried all the parameter combinations, it was automatically adjusted to find the best parameter combination.

#### BP neural network classification model

The BP neural network is supervised learning algorithms in artificial neural networks^20^. It is a multi-layer feedforward network trained according to the error backpropagation algorithm. The BP neural network algorithm also uses Grid Search CV to configure hyperparameters and uses fivefold cross-validation to find the optimal model. The Python toolkit used for this algorithm was the same as that used for the RF algorithm.

#### The fusion models

**Figure 5:**
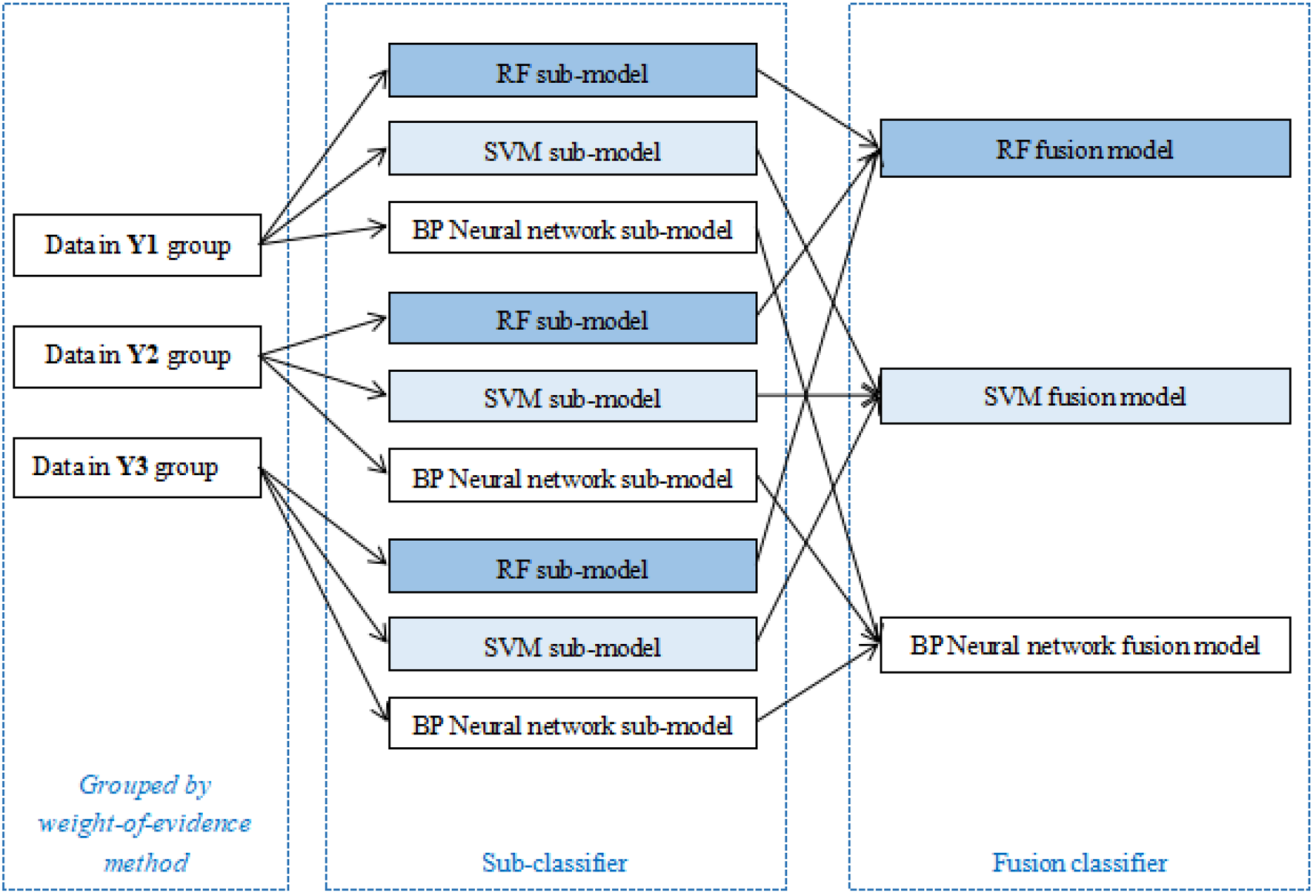
The flowchart of modeling processes.

The final judgement of whether a compound is genotoxic was based on the weight-of-evidence principle: if one or more of the three sub-models under a certain algorithm are positive, the compound is judged as positive for mutagenicity. The generation process of the fused model is shown in Fig. 5.

When the prediction results of the three sub-models are all non-positive, then it is judged negative for mutagenicity. After using three algorithms to predict the groups Y1, Y2, and Y3, according to this principle, the genetic toxicity of the compound was finally determined.

### Model evaluation

Model validation mainly includes two aspects: internal and external validation. The internal verification mainly tests the fitting ability and robustness of the model, and the external test mainly tests the predictive ability of the model.

For validation of QSAR models, fitness is usually evaluated based on the classification prediction accuracy of the training set. The closer the classification accuracy of the training set is to 1, the better the model fit. However, as the number of variables included in the model continues to increase, the fitness will also increase and approximate 1. In such case, the robustness and predictive ability of the model may deteriorate, resulting in overfitting. Therefore, it is necessary to comprehensively evaluate the model in combination with model robustness and predictive ability.

Robustness evaluation is usually carried out in the range of the training set by means of cross-validation (CV)^21,22^. The standard is generally the CV prediction accuracy rate Q. The larger the CV prediction accuracy Q value, the better the stability of the model. This study used fivefold CV.

In the end, the indicators evaluated by the model mainly include overall prediction accuracy; precision, which is the ratio of the number of positive predictions correctly predicted to the total number of positive predictions; recall, that is, the number of positive predictions correctly predicted to the total actual number, the ratio of positive numbers; F1 value (F1-Measure), the weighted harmonic average of precision (precision) and recall (recall). The value is between 0 and 1. The larger the value, the better the model effect. The AUC was calculated from the ROC curve of the test set. The formula was as follows:

1 $${\text{Accuracy}}\;\left( Q \right) = \frac{TP + TN}{{TP + FP + FN + TN}} \times {\text{100\% }}$$

2 $$Precision = \frac{TP}{{(TP + FP)}} \times {\text{100\% }}$$

3 $${\text{Re}} call = \frac{TP}{{(TP + FN)}} \times {\text{100\% }}$$

4 $$F1 = {2} \times \frac{precision \times recall}{{(precision + recall)}} \times {\text{100\% }}$$

where, TP, TN, FP and FN in Eqs. (1)–(4) represent the number of true positive, the number of true negative, the number of false positive and the number of false negative, respectively.

## Supplementary Information

Supplementary Information.

## Data Availability

The results of this study are based on the data from GENE-TOX (https://www.ncbi.nlm.nih.gov/pcsubstance?term=%22Genetic%20Toxicology%20Data%20Bank%20(GENE-TOX)%22%5BSourceName%5D%20AND%20hasnohold%5Bfilt%5D), CPDB(https://www.toxinfo.io/) and CCRIS (https://www.ncbi.nlm.nih.gov/pcsubstance?term=%22Chemical%20Carcinogenesis%20Research%20Information%20System%20(CCRIS)%22%5BSourceName%5D%20AND%20hasnohold%5Bfilt%5D). The data set used in this manuscript can be downloaded through this link ( https://github.com/YangXT-123/Datasets-for-QSAR-research).
