# Supplementary material for: Quantitative structure–activity relationship models for genotoxicity prediction based on combination evaluation strategies for toxicological alternative experiments
Source: Sci Rep. 2021 Apr 13;11:8030. doi: 10.1038/s41598-021-87035-y (PMC8044236; doi:10.1038/s41598-021-87035-y)
Supplement: Supplementary file 1 — Supplementary Information. [file 41598_2021_87035_MOESM1_ESM.docx]

Quantitative Structure-activity Relationship Models for Genotoxicity Prediction Based on Combination Evaluation Strategies for Toxicological Alternative Experiments

Xiaotong Yang^1^, Zhengbao Zhang^2^, Qin Li^2*^, Yongming Cai^3,4*^

^1^ School of Public Health, Guangdong Pharmaceutical University, Guangzhou, China

^2^ Guangdong Province Center for Disease Control and Prevention, Guangzhou, China

^3^ College of Medical information Engineering, Guangdong Pharmaceutical University, Guangzhou, China

^4^ Guangdong Provincial TCM Precision Medicine Big Data Engineering Technology Research Center, Guangzhou, China

**The final PubChem Substructure Fingerprints**

- **Section 1**: Hierarchic Element Counts - These bits test for the presence or count of individual chemical atoms represented by their atomic symbol.
- **Section 2**: Rings in a canonic Extended Smallest Set of Smallest Rings (ESSSR) ring set - These bits test for the presence or count of the described chemical ring system. An ESSSR ring is any ring which does not share three consecutive atoms with any other ring in the chemical structure. For example, naphthalene has three ESSSR rings (two phenyl fragments and the 10-membered envelope), while biphenyl will yield a count of only two ESSSR rings.
- **Section 3**: Simple atom pairs – These bits test for the presence of patterns of bonded atom pairs, regardless of bond order or count.
- **Section 4**: Simple atom nearest neighbors – These bits test for the presence of atom nearest neighbor patterns, regardless of bond order (denoted by "~") or count, but where bond aromaticity (denoted by ":") is significant.
- **Section 5**: Detailed atom neighborhoods – These bits test for the presence of detailed atom neighborhood patterns, regardless of count, but where bond orders are specific, bond aromaticity matches both single and double bonds, and where "-", "=", and "#" matches a single bond, double bond, and triple bond order, respectively.
- **Section 6**: Simple SMARTS patterns – These bits test for the presence of simple SMARTS patterns, regardless of count, but where bond orders are specific and bond aromaticity matches both single and double bonds.
- **Section 7**: Complex SMARTS patterns – These bits test for the presence of complex SMARTS patterns, regardless of count, but where bond orders and bond aromaticity are specific.

| **Section** | **Bit Position** | **Bit Substructure** |
| --- | --- | --- |
| Section 3 | PubchemFP301 | N-O |
| Section 4 | PubchemFP406 | O(~C)(~H) |
| Section 1 | PubchemFP15 | >= 2 N |
| Section 2 | PubchemFP118 | >= 1 saturated or aromatic heteroatom-containing ring size 3 |
| Section 3 | PubchemFP300 | N-N |
| Section 6 | PubchemFP637 | O-C-C-C-C |
| Section 1 | PubchemFP10 | >= 4 C |
| Section 6 | PubchemFP546 | N-C:C-[#1] |
| Section 7 | PubchemFP716 | Cc1ccc(N)cc1 |
| Section 5 | PubchemFP443 | C(-C)(=O) |
| Section 4 | PubchemFP366 | C(~H)(~O) |
| Section 6 | PubchemFP545 | N-C:C-C |
| Section 5 | PubchemFP437 | C(-C)(-N)(=C) |
| Section 2 | PubchemFP115 | >= 1 any ring size 3 |
| Section 2 | PubchemFP178 | >= 1 any ring size 6 |
| Section 1 | PubchemFP56 | >= 1 Cr |
| Section 3 | PubchemFP308 | O-H |
| Section 1 | PubchemFP19 | >= 2 O |
| Section 4 | PubchemFP377 | C(~N)(:C)(:C) |
| Section 4 | PubchemFP385 | C(:C)(:C)(:C) |
| Section 4 | PubchemFP346 | C(~C)(~H)(~O) |
| Section 4 | PubchemFP374 | C(~H)(~H)(~H) |
| Section 2 | PubchemFP200 | >= 4 saturated or aromatic carbon-only ring size 6 |
| Section 7 | PubchemFP734 | Cc1cc(C)ccc1 |
| Section 1 | PubchemFP2 | >= 16 H |
| Section 6 | PubchemFP582 | C-C-C-C-C |
| Section 4 | PubchemFP342 | C(~C)(~Cl) |
| Section 3 | PubchemFP285 | C-N |
| Section 4 | PubchemFP344 | C(~C)(~H) |
| Section 3 | PubchemFP284 | C-C |
| Section 5 | PubchemFP452 | C(-O)(=O) |
| Section 6 | PubchemFP697 | C-C-C-C-C-C(C)-C |
| Section 6 | PubchemFP516 | [#1]-C=C-[#1] |
| Section 2 | PubchemFP185 | >= 2 any ring size 6 |
| Section 1 | PubchemFP9 | >= 2 C |
| Section 1 | PubchemFP37 | >= 1 Cl |
| Section 2 | PubchemFP179 | >= 1 saturated or aromatic carbon-only ring size 6 |
| Section 4 | PubchemFP361 | C(~Cl)(~H) |
| Section 6 | PubchemFP614 | C-C-O-C-C |
| Section 6 | PubchemFP660 | C-C=C-C-C |
| Section 6 | PubchemFP540 | C-N-C-[#1] |
| Section 1 | PubchemFP14 | >= 1 N |
| Section 6 | PubchemFP696 | C-C-C-C-C-C-C-C |
| Section 6 | PubchemFP535 | O=C-C-C |
| Section 3 | PubchemFP283 | C-H |
| Section 3 | PubchemFP286 | C-O |
| Section 4 | PubchemFP390 | N(~C)(~C) |
| Section 1 | PubchemFP11 | >= 8 C |
| Section 1 | PubchemFP12 | >= 16 C |
| Section 1 | PubchemFP39 | >= 4 Cl |
| Section 4 | PubchemFP365 | C(~H)(~N) |
| Section 4 | PubchemFP340 | C(~C)(~C)(~N) |
| Section 6 | PubchemFP567 | O-C-C-O |
| Section 2 | PubchemFP186 | >= 2 saturated or aromatic carbon-only ring size 6 |
| Section 5 | PubchemFP440 | C(-C)(-O)(=O) |
| Section 4 | PubchemFP339 | C(~C)(~C)(~H)(~O) |
| Section 6 | PubchemFP643 | [#1]-C-C-N-[#1] |
| Section 1 | PubchemFP18 | >= 1 O |
| Section 5 | PubchemFP420 | C=O |
| Section 1 | PubchemFP1 | >= 8 H |
| Section 1 | PubchemFP0 | >= 4 H |
| Section 6 | PubchemFP524 | C-C=C-C |
| Section 4 | PubchemFP393 | N(~C)(~H) |
| Section 5 | PubchemFP455 | N(-O)(=O) |
| Section 4 | PubchemFP351 | C(~C)(~N) |
| Section 2 | PubchemFP192 | >= 3 any ring size 6 |
| Section 3 | PubchemFP299 | N-H |
| Section 2 | PubchemFP257 | >= 2 aromatic rings |
